# Evaluation of novel open-source software for cardiac optical mapping

**DOI:** 10.1016/j.jmccpl.2024.100068

**Published:** 2024-03-12

**Authors:** Olivia Baines, Rina Sha, Siddhanth Jatti, Christopher O'Shea

**Affiliations:** Institute of Cardiovascular Sciences, University of Birmingham, Birmingham, UK

**Keywords:** Optical mapping, Software, Analysis

## Abstract

KairoSight-3.0 is a recently released Python-based, open-source software for cardiac optical mapping analysis. Addressing challenges in high-resolution electrophysiological data analysis, KairoSight-3.0 facilitates comprehensive studies of cardiac conduction and excitation-contraction coupling. We compared its performance with ElectroMap, focusing on action potential duration and conduction velocity measurements in mouse heart models subjected to ischaemia and flecainide treatment. Our findings reveal that while both software are effective, inherent methodological differences impact measurement outcomes. KairoSight-3.0's robust analysis capabilities make it a valuable tool in cardiac research. Additionally, future directions for KairoSight-3.0 and other mapping analysis tools are explored.

**Statement of importance:**

Open-source methods for analysis of cardiac optical mapping are vital tools in electrophysiological research. Our work directly evaluates the latest version of KarioSight, recently published in JMCC plus, with ElectroMap, an established and widely used tool. Our results show both software are effective in analysis of changes in both conduction and repolarisation. Considering the new features of KairoSight-3.0 and python implementation, our study importantly demonstrates the effectiveness of the software, highlights potential discrepancies between it and ElectroMap, and provides a perspective on future directions for KairoSight-3.0 and other software.

## 1 Introduction

Optical mapping is a pivotal tool in cardiovascular research, offering high-resolution insights into cardiac electrophysiology across multicellular preparations [1]. Nevertheless, the analysis of optical mapping data presents considerable challenges. The necessity for high spatial and temporal resolution, coupled with inhomogeneous illumination, uneven dye loading, complex conduction patterns, and technical artefacts complicate interpretation and quantification. Further, divergent analysis definitions and approaches exist for several key electrophysiological parameters. As a result, a significant knowledge of signal and image processing, computer science, and cardiac electrophysiology was needed for effective cardiac optical mapping analysis. These factors combined present a barrier to uptake of the approach and objective analysis across different laboratories [2].

However, recent years have seen several powerful tools released for analysis of cardiac optical mapping data [[3], [4], [5], [6], [7]]. These software are important as they are open to all researchers, often packaged into easy-to-use user interfaces, and all methods applied are (to varying degrees) documented and validated. Therefore, previous barriers to effective optical mapping analysis presented by the complex data produced are less prohibitive. Recently, Haq et al. released and manually validated an updated version of the KairoSight-3.0 software, in which they report enhanced capabilities for conduction, duration, alternans, excitation-contraction coupling and extrasystolic (S1-S2) analysis [8]. Importantly, this software is developed in Python, accompanied by detailed install instructions, and so is truly open to all researchers, unlike other options (e.g. MATLAB-based platforms).

Considering the updated and manually validated KairoSight-3.0 version, we sought to compare the use of KairoSight-3.0 to another open-source option for optical mapping analysis, ElectroMap. ElectroMap is a widely utilised MATLAB software for optical mapping analysis that has been validated using experimental and in silico data [3].

## 2 Methods

To perform our comparisons, we used data from a freely available dataset (https://doi.org/10.6084/m9.figshare.c.5700466.v1). Full experimental details are available in the original description of these data [9]. Briefly, mouse whole hearts were isolated, Langendorff perfused and loaded with motion uncoupler Blebbistatin (15 μM) and voltage dye Rh-237 (1.25 mg/ml). Hearts were illuminated at 530 nm, with emitted light collected at >630 nm. Images were collected by an Evolve Delta EMCCD camera, with a sampling frequency of 1 kHz and pixel size of 156 μm. Hearts were paced from the epicardial surface at a pacing cycle length of 110 ms at 4× diastolic threshold for 5 s and recorded throughout. After baseline recordings, 2 interventions were applied i) 1 μM flecainide perfusion and ii) global low-flow ischaemia induced by reduction of flow rate by 75 % for 3 min.

Baseline and treatment data were then analysed using both software, matching processing, and analysis options as closely as possible. 5 × 5 spatial binning was applied, and the same single beat was analysed at the end of the 5 second pacing protocol. Differences in baseline correction, temporal filtering, and multi-beat averaging methods available in the software prevented application of these conditioning approaches.

For conduction velocity (CV) measurement, ‘single vector’ method is the only currently available method in KairoSight-3.0. For these reasons, preliminary studies were performed into variability in CV measurements via single vector method in both software. Activation maps were generated from maximum upstroke time (maximum positive deflection in the optical signal, i.e. dF/dt_max_). Then, in KairoSight-3.0, 15 vectors were placed radially outward from the earliest activation point. In ElectroMap, the semi-automated single vector tool was used to investigate single vector CVs in all directions from a central position.

From these preliminary investigations, it was decided CV would be measured along the manually identified longitudinal direction (direction of fastest CV) with 5 vectors. Minimum and maximum speeds were discarded, and then the mean of the 3 remaining vectors was taken as the conduction speed. For further comparison, multi-vector CV was also used in ElectroMap with default settings [10]. Action potential duration was measured from maximum upstroke time to 80 % repolarisation (APD80).

All results are presented as mean ± standard error, with differences between treatment or software examined by paired Student's *t*-test or one-way ANOVA as appropriate.

## 3 Results

**Fig. 1:**
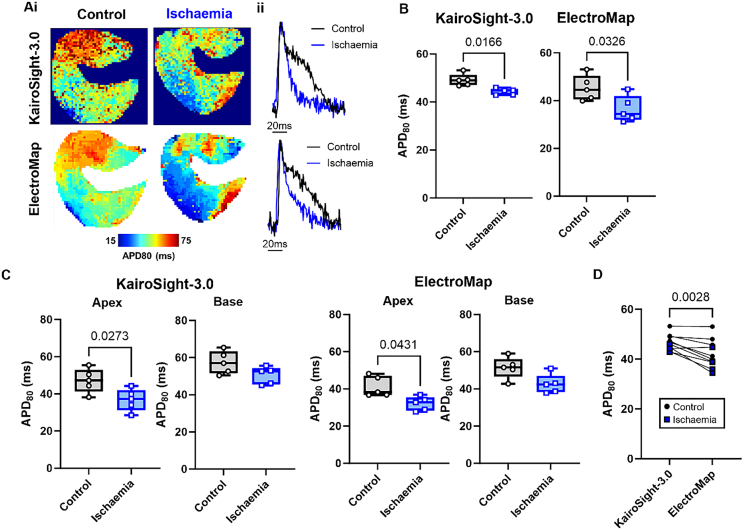
Action potential measurements. A) Example APD80 maps (i) and action potential signals (ii) in control and ischaemic conditions as measured by KairoSight-3.0 (top panels) and ElectroMap (bottom panels). B) Grouped APD80 data (mean from entire imaged surface) showing changes in APD80 during ischaemia as measured by KairoSight-3.0 and ElectroMap. *n* = 5 hearts. C) Regional grouped APD80 data from either the apex or base of the heart showing changes in APD80 during ischaemia as measured by KairoSight-3.0 and ElectroMap. *n* = 5. D) Comparison of APD80 values as measured by KairoSight-3.0 and ElectroMap during control (black circles) and ischaemic (blue squares) *n* = 10 hearts, comparisons made across both control and ischaemic conditions. Error bars show standard maximum to minimum. All group differences tested by paired *t*-test, with *p* < 0.05 level of significance. (For interpretation of the references to colour in this figure legend, the reader is referred to the web version of this article.)

Ischaemia causes several effects on myocardial tissue including increased concentration of potassium in the extracellular space and strong activation of K_ATP_ channels, contributing to stronger repolarisation currents and so shortening of action potential duration [11]. Fig. 1A demonstrates global ischaemia shortens APD80 as measured by KairoSight-3.0 (49.1 ± 1.1 to 44.3 ± 0.6 ms, *p* = 0.017, *n* = 5) and ElectroMap (45.3 ± 2.3 to 36.5 ± 2.5 ms, *p* = 0.032, n = 5), Fig. 1B. Ischaemia asserted similar effects on APD80 in the base and apex of the left ventricle although significance was lost in the base, as measured by both software, Fig. 1C. APD80 tended to be shorter when measured in ElectroMap compared to KairoSight-3.0 (42.1 ± 1.8 vs 46.7 ± 1.1 ms, ElectroMap vs KairoSight-3.0, *p* = 0.028, *n* = 10), with greater inter-experiment variability, Fig. 1D.

**Fig. 2:**
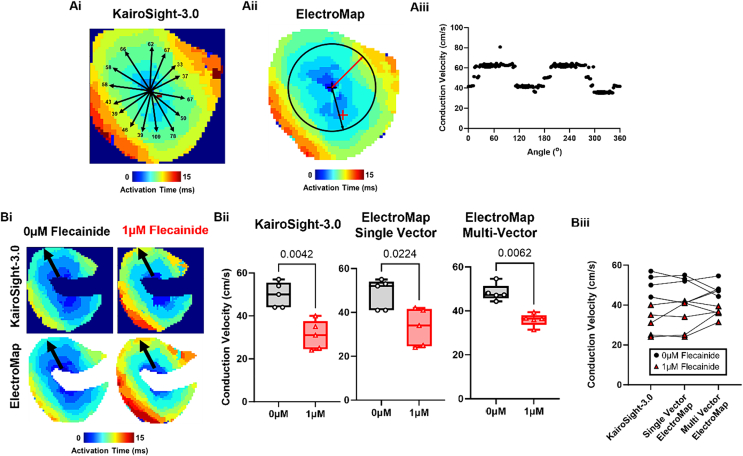
Conduction velocity measurements. Ai) Example activation map from KairoSight-3.0 with superimposed conduction vectors. Black text shows measured conduction speed (in cm/s). Aii) Example activation map from ElectroMap circular radius from which 360 (one per degree) single vector conduction speeds were calculated. Red and black velocity vectors show time of slowest and fastest measured conduction respectively. Aiii) Angular profile of measured conduction speeds in ElectroMap. Bi) Example activation maps before and after 1 μM flecainide treatment as measured by KairoSight-3.0 (top panels) and ElectroMap (bottom panels). Black arrows indicate direction of longitudinal conduction. Bii) Grouped conduction velocity data showing changes in conduction velocity induced by 1 μM flecainide as measured by KairoSight-3.0 and ElectroMap (single vector) and additionally using ElectroMap multi-vector method, n = 5 hearts. Effect of flecainide treatment was tested by paired t-test, with p < 0.05 level of significance. Biii) Comparison of conduction velocity values as measured by KairoSight-3.0 and ElectroMap during control (black circles) and 1 μM flecainide treatment (red triangles), n = 10 hearts. Effect of software and method treatment was tested by One-way ANOVA with p < 0.05 level of significance. Error bars show maximum to minimum. (For interpretation of the references to colour in this figure legend, the reader is referred to the web version of this article.)

Potential variability in single vector measured CV was investigated using both software, Fig. 2A. In KairoSight-3.0, 15 manual vectors along similar distances from the earliest activation point yielded CV measurements ranging from 33 to 109 cm/s. Using the semi-automated single vector measurement feature in ElectroMap, CV values ranged from 38 to 81 cm/s.

Flecainide is a sodium channel blocker that reduces the inward sodium current and hence is expected to reduce myocardial conduction speed [12]. Fig. 2Bii shows that 1 μM flecainide indeed slows CV as measured by KairoSight-3.0 (49.8 ± 2.6 to 31.0 ± 3.1 cm/s, *p* = 0.042, *n* = 5), ElectroMap single vector (48.4 ± 3.1 to 33.2 ± 3.8 cm/s, *p* = 0.022, n = 5) and ElectroMap multi-vector (48.3 ± 1.7 to 35.6 ± 1.3 cm/s, *p* = 0.0062, n = 5). There were no systematic differences between CV dependent on software or method, Fig. 2Biii.

## 4 Discussion

Taken together, these results show how both software are effective in measuring changes in action potential duration and conduction velocity. However, there are some differences between duration measurements which can be attributed to the different methodologies employed by both software. These results, combined with robust validation, extra features, and Python implementation, demonstrate KairoSight-3.0 as useful tool for optical mapping analysis.

Open-source software such as KairoSight-3.0 will continue to evolve. For example, future implementation of multi-vector and angular conduction velocity measurements within KairoSight-3.0 will expand the tools available to analyse cardiac conduction, arguably one of the most important uses of optical mapping in cardiac research. Current single-vector implementation successfully measured flecainide conduction slowing herein and has several advantages including direct user guided comparison of longitudinal and transverse conduction. It also enables a user to avoid areas where CV measurement will be erroneous, such as areas of pacing artefacts or transmural breakthrough.

However, there are also limitations with respect to the potential for user bias and the sensitivity of the technique to point placement. Fig. 2A demonstrates that the large range of single vector CVs that can be yielded even when measured along similar distances from the same activation point. The reasons for this variability are multifaceted, including conduction dynamics (e.g. whether a vector is along longitudinal or transverse direction), sample rate limited activation definitions reducing effective resolution, and artefacts induced by epicardial pacing site and surface visualisation of transmural conduction. However, these analyses demonstrate the inherent variability and sensitivity to user selection that can highly influence CV measurements in cardiac tissue when measured between 2 points.

Notably, while APD measurements with both KairoSight-3.0 and ElectroMap showed expected treatment effects, discrepancies were observed. KairoSight-3.0 produced longer APDs and less inter-experiment variability. Despite efforts to align processing and analysis, inherent software differences, particularly in signal interpolation and baseline definition, led to variations in APD measurements. This highlights the impact of software design on data interpretation and the need for customisable settings like baseline definition. Therefore, an understanding of signal quantification methods and cardiac electrophysiology is still needed for accurate analysis.

Moreover, these findings underscore the critical role of ‘ground-truth’ validation, such as employing modelled datasets, in ensuring the reliability of measured parameters. KairoSight-3.0 has already been validated against manually annotated datasets [8], while in ElectroMap development some simple in silico modelling has been performed [3]. These validation studies must be expanded however in new software developments. For example, observed reduction in inter-experiment variability might indicate that KairoSight-3.0 provides more consistent duration measurements compared to ElectroMap under the current settings. However, to validate these observations comprehensively, further evaluations should be conducted using in silico methods with known variation across datasets.

### 4.1 Future directions for open-source optical mapping analysis

Here we have only compared two optical mapping software. However, several more are available to the community, each with specific strengths and capabilities. Some notable examples include COSMAS [5], which shows comparable or better results than ElectroMap for some important analyses with a simpler codebase and Python implementation (but with no user interface), and RHYTHM whose latest versions include handling of 3D panoramic imaging [2,6]. Despite these tools however, there are several areas which future software can be expanded in to improve optical mapping analysis.

There is a need for software to achieve multi-parametric analysis. This extends beyond dual analysis of voltage and calcium signals as already possible with KairoSight-3.0 and other software. Potentially key insights are available from correlating electrophysiological parameters with optical signals (either through indicators or autofluorescence) for several important molecules (e.g. NADH, cAMP, reactive oxygen species) and/or membrane potential changes of organelles such as the mitochondria [13,14].

Reliable signal to noise ratio qualification is required. Although both KairoSight-3.0 and ElectroMap include signal to noise quantification options, they are not without limitation. Methods employed by both systems rely on a diastolic period to compute noise levels for comparison with signal amplitude. This is limited in cases where no such discernible period exists (e.g. at fast pacing cycle lengths). Therefore, implementation of more robust or at least tuneable signal to noise ratio qualification methods is needed.

Most software is understandably designed to deal with sinus rhythm/paced data. This is due to several factors, paramount amongst which is the difficulty in quantifying inherently disordered and low amplitude signals derived from optical recordings taken during arrhythmia. Some specialised tools for this purpose are available, for example for tracking of phase singularities during fibrillation [7], and tools such as ElectroMap incorporate simple approaches such as dominant frequency analysis and phase mapping [3]. However, further development of tools for quantifiable analysis of optical recordings during arrhythmia may yield new insights into arrhythmia mechanisms and termination.

A longstanding limitation of optical mapping has been the requirement for motion uncoupling (stopping the mechanical contraction of the heart while preserving electrical excitation). This is mostly achieved through electromechanical uncouplers such as Blebbistatin to prevent motion artefacts in recorded optical signals. Some evidence however has shown compounds such as Blebbistatin alter electrophysiology. Although this is debated, uncoupling undoubtably omits physiologically important bidirectional electromechanical interactions and alters metabolic demand. Recently however, several groups have reported optical mapping in the freely beating heart (and 2D and 3D cell models) made possible by sophisticated computational methods such as optical flow [15,16]. Although computationally sophisticated, these methods are often based on open-source libraries for their application [17]. Therefore, integration of these approaches in available software would remove one important barrier to optical mapping of the freely beating heart to the community.

For several of these developments, artificial intelligence (AI) techniques may play a key role. AI automated signal morphology classification could have several uses in open-source software. This includes automatic recognition of experimental model (e.g. mouse, rabbit, pig, human etc.) or recorded signal (e.g. voltage, calcium etc.) to optimise processing and analysis settings without user input. AI based techniques for automated artefact detection has several potential applications. Such an approach could help distinguish between motion artefacts and true (patho-)physiological phenomena such as early or delayed afterdepolarisations. Equally, it could be used as a tool to remove areas of the tissue corrupted by artefacts, or indeed decide where application of motion tracking techniques described above is required.

In conclusion, the development and refinement of tools such as KairoSight-3.0 are vitally important for cardiac electrophysiological research and application of optical mapping. KairoSight-3.0 features enhance capabilities, and Python-based deployment makes it accessible to researchers across the field. Further development of KairoSight-3.0 and other software will continue to expand the use of optical mapping to advancing our understanding of cardiac function and disease.

## Funding statement

The authors are supported by the 10.13039/100010269Wellcome Trust (221650/Z/20/Z); 10.13039/501100000274British Heart Foundation, (FS/PhD/22/29309) and British Heart Foundation Accelerator Award to the Institute of Cardiovascular Sciences, 10.13039/501100000855University of Birmingham (AA/18/2/34218).

## Use of generative AI

During the preparation of this work the authors used ChatGPT 4.0 to copy edit and improve on original text drafted de novo by the authors. After using this tool, the authors reviewed and edited the content as needed and take full responsibility for the content of the publication.

## CRediT authorship contribution statement

**Olivia Baines:** Writing – original draft, Investigation, Formal analysis. **Rina Sha:** Writing – review & editing, Investigation, Formal analysis. **Siddhanth Jatti:** Data curation, Formal analysis, Writing – review & editing. **Christopher O'Shea:** Writing – original draft, Software, Funding acquisition, Formal analysis, Data curation, Conceptualization.

## Declaration of competing interest

The authors declare that they have no competing interests.
